# Epidemiological Analysis of Multidrug-Resistant *Acinetobacter baumannii* Isolates in a Tertiary Hospital Over a 12-Year Period in China

**DOI:** 10.3389/fpubh.2021.707435

**Published:** 2021-08-12

**Authors:** Meijie Jiang, Xia Chen, Shuang Liu, Zhijun Zhang, Ning Li, Chao Dong, Ling Zhang, Haiyan Wu, Shuping Zhao

**Affiliations:** ^1^Tai'an City Central Hospital, Taian, China; ^2^College of Animal Science and Veterinary Medicine, Sino-German Cooperative Research Centre for Zoonosis of Animal Origin Shandong, Shandong Provincial Key Laboratory of Animal Biotechnology and Disease Control and Prevention, Shandong Provincial Engineering Technology Research Center of Animal Disease Control and Prevention, Shandong Agricultural University, Taian, China

**Keywords:** multidrug-resistant *Acinetobacter baumannii*, resistance genes, sequence types, molecular epidemiology, genetic evolution

## Abstract

*Acinetobacter baumannii* is an important nosocomial pathogen, which is multidrug resistant (MDR). *Acinetobacter baumannii* has become a major threat to public health worldwide due to its ability to easily acquire resistant genes. In order to analyze its epidemiology characteristics and the genetic evolution, *A. baumannii* isolates obtained from a Chinese tertiary hospital in the past 12 years (2008–2019), 295 isolates of non-repetitive *A. baumannii*, were recovered from patients and wards environments. The resistance genes were analyzed using antimicrobial susceptibility testing. The genetic relatedness of 295 isolates was identified by multilocus sequence typing (MLST) and eBURST analysis. It was found that the antibiotic-resistant and carbapenemase-resistant genes of all the 295 MDR *A. baumannii* in the hospital have not changed significantly over the past 12 years; all of them were resistant to multiple antibiotics except the polymyxin E and tigecycline. The results of drug-resistant genes showed that the detection rates of carbapenemase-resistant genes *bla*_*OXA*−23_, *bla*_*TEM*−1_, and *bla*_*OXA*−66_ were 97.6, 75.3, and 71.9%, respectively, which were detected almost every year from 2008 to 2019. Additionally, 16s rRNA methylation enzyme gene *armA*, aminoglycoside-resistant gene *ant(3")-I*, and class I integrase gene could also have a high positive rate. By MLST, these isolates were assigned to 12 sequence types (STs), including ST369, ST208, ST195, ST191, ST368, ST530, ST469, ST451, ST229, ST381, ST543, and ST1176. eBURST analysis showed that 9 STs with ST208 as the founder genotype belonged to Group 1 except for ST229, ST530, and ST1176. Therefore, most MDR *A. baumannii* isolates had a relatively close genetic relationship. Notably, the predominant ST208 and ST369 at the early stage changed to ST451 in 2019, indicating that the complex and diverse genetic background of the prevalence of *A. baumannii* isolates in the hospital. Overall, further epidemiological surveillance and genetic evolution analysis of *A. baumannii* are required, which can provide new strategies for the prevention and control of *A. baumannii* infections.

## Introduction

*Acinetobacter baumannii*, a Gram-negative and non-fermentative bacterium, is an important opportunist pathogen in hospitals. It can cause a wide range of severe nosocomial infections, including ventilator-associated pneumonia, bloodstream infections, skin and soft tissue infections, wound infections, urinary tract infections, and meningitis (1).

*Acinetobacter baumannii* is found almost exclusively in the hospital environment; it can easily colonize the skin surface, respiratory tract, and digestive tract of patients (2). Moreover, another concern for *A. baumannii* is the drug resistance. A large number of studies showed that *A. baumannii* presents resistance to multiple antimicrobial agents, including carbapenems, and multidrug resistance (MDR) is very common (3). Recently, extensively drug-resistant (XDR) and pandrug-resistant (PDR) *A. baumannii* isolates have rapidly increased (4). Therefore, the World Health Organization (WHO) has assigned *A. baumannii* as a critical priority pathogen posing a great threat to public health, and toward which new antibiotics are urgently needed.

Although there are a good deal of studies on the outbreaks caused by *A. baumannii* worldwide, medical environments and drug and disinfection strategies for *A. baumannii* infection are different in different regions and hospitals, leading to the different selective pressures on this bacteria; as a result, there are certain differences in bacterial dominance types and resistance. In the present study, *A. baumannii* isolates over the past 12 years (2008–2019) have been analyzed for the molecular epidemiology and evolution characteristics in a tertiary hospital in Shandong province, China. The epidemiology analysis of *A. baumannii* is helpful for understanding its genetic variation and providing insights into the treatment and control of this bacterial infection.

## Materials and Methods

The study was carried out in accordance with the approved guidelines of the Ethics Committee of Taian City Central Hospital with written informed consent from all subjects. All the subjects gave a written informed consent in accordance with the *Declaration of Helsinki*.

### Acinetobacter Baumannii Isolates

Two hundred and ninety-five isolates of non-repetitive *A. baumannii* were isolated from clinical samples collected during a routine checkup by medical professionals and the wards environments from October 2008 to October 2019. During the 12 years period, 195 isolates were recovered from the sputum, 86 isolates were from the wards environments, and 9, 3, and 2 isolates were from cerebrospinal fluid, wound, and urine, respectively. These samples were collected during a period when *A. baumannii* was relatively prevalent clinically. As for wards distribution, most isolates (64.7%) were collected from intensive care unit (ICU), and 29.2% isolates were from ICU environments. A small number of isolates was from other wards, including nephrology ward (NW), health care ward (HCW), and cardiology ward (Table 1). On the whole, 295 isolates were from patients (sputum, cerebrospinal fluid, and urine) and wards environments, including pillow slips, quilts, stethoscopes, infusion pumps, ventilators, monitors, nurse/doctor cuffs, wristbands, blood pressure monitors, bedside tables, and others used by patients. The detailed isolation information of MDR *A. baumannii* isolates from 2018 and 2019 are shown in Supplementary Table 1.

**Table 1:** The source and distribution of 295 *A. baumannii* isolates recovered from 2008 to 2019.

**Periods**	**The number of isolates**	**Sample sources** ^**a**^					**Wards distribution** ^**b**^													
		**SP**	**CF**	**WO**	**UR**	**IE**	**ICU1**	**ICU2**	**ICU3**	**ICU4**	**ICU5**	**NW**	**HCW**	**CW**	**PW**	**EW**	**NSW**	**GW**	**ICU1-E**	**ICU3-E**
2008.10–2009.2	10	10					10													
2010.6–2011.6	46	44	1	1			16	11	6	11	2									
2012.12–2013.10	42	39	1	1	1		11	4	7	10	4		1	1	1	3				
2014.1–2014.11	41	23		1		17	12	3	7				2						3	14
2015.10–2016.2	55	54	1				20	6	18	5	2		2	1	1					
2017.2–2017.10	15	12	2		1		7	3	1		2	1					1			
2018.2–2018.11	27	4	4			19	2		2								2	2	9	10
2019.2–2019.10	59	9				50			9											50
Sum.	295	195	9	3	2	86	78	27	50	26	10	1	5	2	2	3	2	3	12	74

a *SP, Sputum; CF, Cerebrospinal fluid; WO, Wound; UR, Urine; IE, ICU environment*.

b *ICU, Intensive care unit; NW, Nephrology ward; HCW, Health care ward; CW, Cardiology ward; PW, Pediatrics ward; EW, Emergency ward; NSW, Neurosurgery ward; GW, Gastroenterology ward; ICU1-E, ICU1 environment; ICU3-E, ICU3 environment*.

### Bacterial Identification and Antimicrobial Susceptibility Testing

Bacterial identification was performed by WalkAway 96 PLUS-NC50 combo panel (Beckman, United States) following the instructions of the manufacturer. Antimicrobial susceptibility testing was performed by three different methods: the sensitivity of meropenem and cefotaxime was determined by the disk diffusion method, the sensitivity of tigecycline and polymyxin E was determined by the Etest method (AB Biodisk, Solna, Sweden), and the sensitivity of other antimicrobial agents was detected using the WalkAway 96 PLUS-NC50 combo panel. The criteria of the susceptibility of the PLUS-NC50 combo panel, polymyxin E, meropenem, and cefotaxime were adapted from the Clinical and Laboratory Standards Institute (CLSI; http://clsi.org/standards/). The criteria of the susceptibility of tigecycline were adapted from the U. S. Food and Drug Administration (http://www.fda.org.uk/sitemap.aspx).

### Detection of Antimicrobial-Resistant Genes

Bacterial DNAs were extracted and the primers of the related resistance genes were designed as described previously (5–8), including carbapenemase-resistant genes *bla*_*OXA*−23_, *bla*_*OXA*−24_, *bla*_*OXA*−48_, *bla*_*OXA*−50_, *bla*_*OXA*−58_, *bla*_*OXA*−60_, *bla*_*OXA*−66_, *bla*_*OXA*−197_, *bla*_*KPC*_, *bla*_*TEM*−1_, *bla*_*NDM*−1_, and *bla*_*IMP*−4_; the 16s rRNA methylase-resistant genes *armA*; and the aminoglycoside-resistant genes *ant(3")-I, aac(3)-I, aac(3)-II, aac(6*′*)-I, aac(6*′*)-II*, and *aph(3*′*)-VI*. In addition, the detection primers of integrases and integron genes were designed and synthesized, the specific sequences are shown in Table 2.

**Table 2:** The detection primers of integrase and integron genes.

**Gene names**	**Primer sequence (5^**′**^ → 3^**′**^)**	**Product size (bp)**
Class I integrase gene	P1: CCGAGGATGCGAACCACTTC	373
	P2: CCGCCACTGCGCCGTTACCA	
Class II integrase gene	P1: CACGGATAGCGACAAAAAGGT	789
	P2: GTAGCAAACGAGTGACGAAATG	
Class III integrase gene	P1: GCCTCCGGCAGCGACTTTCAG	433
	P2: GATGCTGCCCAGGGCGCTCG	
Class I integron variable	P1: GGC ATC CAA GCA GCA AG	Unknown
region	P2: AAG CAG ACT TGA CCT GA	

### Multilocus Sequence Typing

Seven housekeeping genes (*gltA, gyrB, gdhB, recA, cpn60, gpi, rpoD*) were amplified and sequenced to determine the genotypes of all isolates. DNA sequence variations and sequence types (STs) were analyzed using the multilocus sequence typing (MLST) database for *A. baumannii* (http://pubmlst.org/abaumannii). MLST was performed using the Oxford scheme as previously described (9), and eBURST method was used for the analysis of the novel alleles and genetic evolution (http://www.phyloviz.net/goeburst/).

## Results

### Susceptibility Testing

The sensitivity of all *A. baumannii* isolates recovered from 2008 to 2019 to 15 antimicrobial agents was shown in Table 3, all of them exhibited an MDR phenotype, being resistant to three or more classes of antibiotics, such as amikacin, gentamicin, ceftazidime, ceftriaxone, piperacillin/tazobactam, imipenem, meropenem, levofloxacin, and ciprofloxacin, and the resistance of isolates to these drugs has not changed much over the past 12 years. However, all 295 MDR *A. baumannii* isolates were sensitive to polymyxin E, indicating that this antibiotic had a good therapeutic effect on *A. baumannii* in the hospital. In addition, these isolates were highly sensitive to tigecycline, although the sensitivity declined during the years 2010–2013 (59.5–60.9%) and 2015–2016 (74.5%).

**Table 3:** The antimicrobial susceptibility testing of *A. baumannii* isolates to 15 antibiotics over the past 12 years.

**Antibiotics**	**2008–2009** **(*n* = 10)**	**2010–2011** **(*n* = 46)**	**2012–2013** **(*n* = 42)**	**2014** **(*n* = 41)**	**2015–2016** **(*n* = 55)**	**2017** **(*n* = 15)**	**2018** **(*n* = 27)**	**2019** **(*n* = 59)**
	**Susceptibility**	**Susceptibility**	**Susceptibility**	**Susceptibility**	**Susceptibility**	**Susceptibility**	**Susceptibility**	**Susceptibility**
	**[*n* (%)]**	**[*n* (%)]**	**[*n* (%)]**	**[*n* (%)]**	**[*n* (%)]**	**[*n* (%)]**	**[*n* (%)]**	**[*n* (%)]**
Amikacin	0	5 (10.9%)	22 (53.4%)	2 (8.0%)	1 (1.8%)	2 (13.3%)	1 (3.7%)	4 (6.7%)
Gentamicin	0	2 (4.3%)	0	2 (8.0%)	1 (1.8%)	0	1 (3.7%)	4 (6.7%)
Tobramycin	0	5 (10.9%)	22 (53.4%)	2 (8.0%)	1 (1.8%)	1 (6.7%)	1 (3.7%)	4 (6.7%)
Ceftazidime	0	0	0	0	0	0	0	0
Ceftriaxone	0	0	0	0	0	0	0	0
Cefepime	0	0	0	0	0	0	0	0
Piperacillin/tazobactam	0	0	0	1 (4.0%)	0	0	0	0
Cefoperazone/sulbactam	0	6 (13.0%)	1 (4.8%)	1 (4.0%)	3 (5.5%)	0	0	0
Meropenem	0	0	1 (2.4%)	1 (4.0%)	0	0	0	0
Imipenem	0	0	1 (2.4%)	1 (4.0%)	0	0	0	0
Levofloxacin	0	0	0	2 (8.0%)	0	5 (33.3%)	0	0
Ciprofloxacin	0	0	0	2 (8.0%)	0	0	0	0
Cotrimoxazole	0	1 (2.2%)	0	0	2 (3.6%)	0	8 (29.6%)	2 (3.4%)
Tigecycline	10 (100%)	28 (60.9%)	25 (59.5%)	41 (100%)	41 (74.5%)	15 (100%)	27 (100%)	59 (100%)
Polymyxin E	10 (100%)	46 (100%)	42 (100%)	41 (100%)	55 (100%)	15 (100%)	27 (100%)	59 (100%)

### Drug-Resistant Genes

As many as 288 isolates (97.6%) carried *bla*_*OXA*−23_ gene, followed by *bla*_*TEM*−1_ (75.3%) and *bla*_*OXA*−66_ (71.9%) genes, which were detected almost every year from 2008 to 2019. The drug-resistant gene *bla*_*OXA*−197_ was detected in 28 isolates in 2014, *bla*_*NDM*−1_and *bla*_*IMP*−4_ genes were only detected in 2010–2011, the corresponding numbers of isolates were 1 and 6, respectively (Table 4). Other carbapenemase genes were not detected in any of these isolates. The 16s rRNA methylation enzymes gene *armA* can be detected every year, and the total 250 isolates carried this resistant gene over the past 12 years (Table 5). The aminoglycoside-resistant gene *ant(3")-I* can also be detected every year, and the number of *A. baumannii* isolates harboring this gene was the most, accounting for 88.1%, followed by *aac(6")-I* (41.7%) and *aac(3)-I* (32.2%). However, all *A. baumannii* isolates carrying *aac(3)-I* gene appeared before 2016, and *aac(6")-I* was predominant in *A. baumannii* isolates from 2017 to 2019 (77.2%) (Table 6).

**Table 4:** The profiles of the carbapenemase genes of 295 MDR *A. baumannii* isolates.

**Periods**	**The number of isolates**	**Carbapenemase genes**					
		***bla_**OXA-23**_***	***bla*_**OXA-66**_**	***bla*_**OXA-197**_**	***bla_**NDM-1**_***	***bla_**IMP-4**_***	***bla_**TEM-1**_***
2008–2009	10	+	+	−	−	−	−
2010–2011	1	+	+	−	+	+	−
	5	−	+	−	−	+	−
	40	+	+	−	−	−	−
2012–2013	1	−	+	−	−	−	+
	34	+	+	−	−	−	+
	7	+	+	−	−	−	−
2014	28	+	−	+	−	−	+
	7	+	+	−	−	−	+
	5	+	+	−	−	−	−
	1	−	+	−	−	−	+
2015–2016	50	+	−	−	−	−	+
	5	+	−	−	−	−	−
2017	15	+	+	−	−	−	+
2018	27	+	+	−	−	−	+
2019	59	+	+	−	−	−	+
Sum.	295	288	212	28	1	6	222

“*+” indicates the carbapenemase gene was detected; “−” indicates the carbapenemase gene was not detected; “S” indicates the isolate was susceptible to the antibiotic; “R” indicates the isolate was resistant to the antibiotic*.

**Table 5:** The profiles of the 16s rRNA methylation enzymes gene of 295 MDR *A. baumannii isolates*.

**Periods**	**The number of isolates**	**16s rRNA methylation enzymes gene *armA***
2008–2009	10	+
2010–2011	40	+
	6	−
2012–2013	16	+
	26	−
2014	36	+
	5	−
2015–2016	54	+
	1	−
2017	13	+
	2	−
2018	26	+
	1	−
2019	55	+
	4	−
Sum.	295	250

**Table 6:** The profiles of the aminoglycoside resistant genes of 295 MDR *A. baumannii* isolates.

**Periods**	**The number of isolates**	**Aminoglycoside resistant genes**					
		***ant(3")-I***	***aac(3)-I***	***aac(6^**′**^)-I***	***aac(3)-II***	***aac(6^**′**^)-II***	***aph(3^**′**^)-VI***
2008–2009	5	+	−	+	−	−	−
	2	−	−	−	−	−	−
	2	+	+	−	−	−	−
	1	+	−	−	−	−	−
2010–2011	31	+	+	−	−	−	−
	6	+	−	−	−	−	−
	1	+	−	−	−	−	−
	1	+	+	−	+	−	+
	1	+	−	−	−	+	−
	1	+	+	−	+	−	−
	5	−	−	−	−	−	−
2012–2013	33	+	+	−	−	−	−
	6	+	−	+	−	−	−
	1	+	+	+	−	−	−
	1	+	−	−	−	−	−
	1	−	−	−	−	−	−
2014	27	+	−	−	−	−	−
	5	+	+	−	−	−	−
	7	−	+	−	−	−	−
	2	−	−	−	−	−	−
2015–2016	2	+	+	+	−	−	−
	12	+	+	−	−	−	−
	13	+	−	+	−	−	−
	16	+	−	−	−	−	−
	1	−	−	+	−	−	−
	11	−	−	−	−	−	−
2017	14	+	−	+	−	−	−
	1	−	−	−	−	−	−
2018	26	+	−	+	−	−	−
	1	−	−	−	−	−	−
2019	55	+	−	+	−	−	−
	4	−	−	−	−	−	−
Sum.	295	260	95	123	2	1	1

Furthermore, Classes I, II, and III integrase genes of several MDR *A. baumannii* isolates from 2014 to 2019 (197 isolates) were detected. The results showed that 170 isolates carried Class I integrase gene (Intl1), with a positive rate of 86.3%, but none of them contained Classes II and III integrase genes. The PCR-positive products of eight isolates of Class I integrase gene were sequenced and confirmed its accuracy. Additionally, we further detected the integron variable regions of some MDR *A. baumannii* isolates that were positive for class I integrase genes. It was found that there were no drug-resistant genes in the 500, 750, and 1,000 bp segments, while the 1,500–2,200 bp segments contained *aacC1, aadA1, aacA4, catB8*, and *arr3* genes. Among them, *aacA4* and *aadA1* were aminoglycoside-resistant genes, and *catB8* was a chloramphenicol-resistant gene. These results indicated that the Class I integrase genes of MDR *A. baumannii* isolates from this hospital may mainly mediate aminoglycoside and chloramphenicol resistance.

### Multilocus Sequence Typing

A total of 12 STs were detected for the 295 MDR *A. baumannii* isolates by MLST molecular typing, including ST369, ST208, ST195, ST191, ST368, ST530, ST469, ST451, ST229, ST381, ST540, and ST1176. As shown in Table 7, ST208 was predominant in all *A. baumannii* isolates (30.8%); then ST451 (27.1%), ST369 (17.6%), ST195 (12.9%), and ST368 (6.1%); and the remaining STs were few, with 1–6 isolates. Notably, the STs of all *A. baumannii* isolates from 2008 to 2019 showed a certain change. In 2010–2016, the number of ST208 was highest (44.8%, 87/194) and the isolation rate of ST208 in this period accounted for 95.6% (87/91) of the total ST208 in 12 years, especially it was predominant in 2010–2013. During 2014–2016, the prevalence of ST369 gradually increased. In 2017–2018, ST195 was the dominant ST in the hospital, but in 2019, ST451 was predominant. These results demonstrated that the predominant STs of *A. baumannii* isolates are different in different periods in the hospital, indicating the diverse and complicated genetic background of *A. baumannii* isolates in the hospital.

**Table 7:** The MLST result of 295 MDR *A. baumannii* isolates.

**Periods**	**The number of isolates**	**MLST type**											
		**ST**	**ST**	**ST**	**ST**	**ST**	**ST**	**ST**	**ST**	**ST**	**ST**	**ST**	**ST**
		**369**	**208**	**195**	**191**	**368**	**530**	**469**	**451**	**229**	**381**	**540**	**1,176**
2008–2009	10	2	2	1	−	2	−	2	−	1	−	−	−
2010–2011	46	2	24	−	−	5	−	4	7	−	4	−	−
2012–2013	42	6	29	−	−	7	−	−	−	−	−	−	−
2014	41	23	13	4	−	−	1	−	−	−	−	−	−
2015–2016	55	14	21	11	−	2	−	−	6	−	1	−	−
2017	15	3	2	7	−	2	−	−	−	−	−	1	−
2018	27	2	−	15	−	−	−	−	9	−	−	−	1
2019	59	−	−	−	1	−	−	−	58	−	−	−	−
Sum.	295	52	91	38	1	18	1	6	80	1	5	1	1

**Figure 1:**
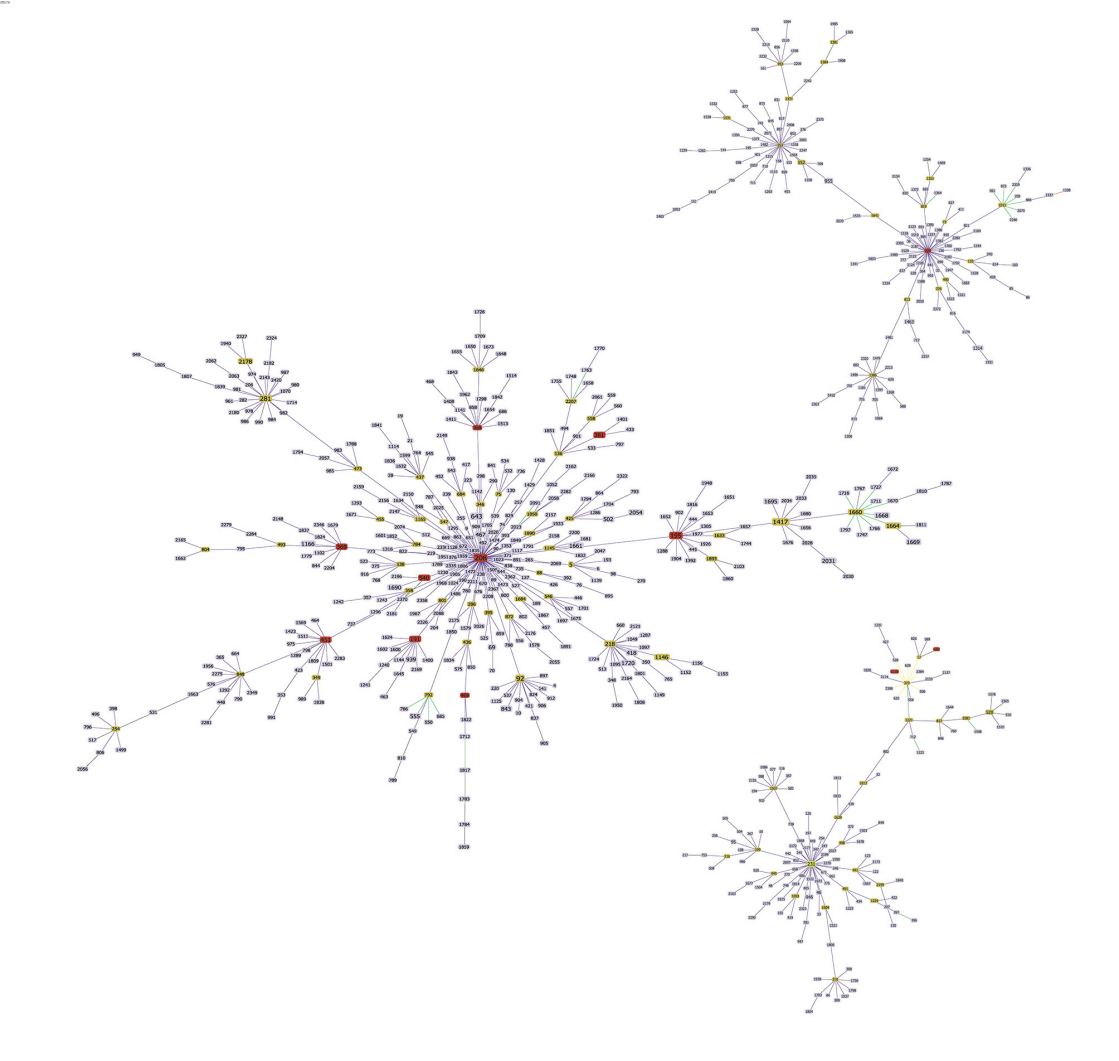
eBURST analysis of 295 MDR *Acinetobacter baumannii* isolates. It was found that these 12 STs could be divided into three groups. ST369, ST208, ST195, ST191, ST368, ST469, ST451, ST381, and ST540 belonged to Group 1, and ST208 was the central type. ST229 belonged to Group 2, and ST530 and ST1176 constituted Group 3.

In order to analyze the genetic evolution of 295 MDR *A. baumannii* isolates, eBURST method was performed. As was shown in Figure 1, these 12 STs could be divided into three groups. ST369, ST208, ST195, ST191, ST368, ST469, ST451, ST381, and ST540 belonged to Group 1, with the founder genotype ST208. ST229 belonged to Group 2, and ST530 and ST1176 constituted Group 3. Therefore, most MDR *A. baumannii* isolates obtained in this study were of Group 1, and they had relatively close genetic relationship. Moreover, some representative isolates were selected for pulse field gel electrophoresis analysis (Supplementary Figure 1), and it was found that MDR *A. baumannii* isolates with the same ST and obtained from the same year were not exactly the same clone, indicating the relative complexity of prevalent MDR *A. baumannii* isolates in this hospital.

## Discussion

*Acinetobacter baumannii* has recently been considered the most critical pathogen for posing a great threat to public health. In order to systematically summarize and investigate the prevalence and genetic evolution of *A. baumannii* and prevent the outbreak and patient-infections caused by MDR *A. baumannii*, we retrospectively analyzed the molecular epidemiology characteristics of 295 *A. baumannii* isolates in a tertiary teaching hospital for 12 years (2008–2019), including drug resistance, drug-resistant genes, and STs. In the present study, 295 *A. baumannii* isolates were mainly recovered from sputum, cerebrospinal fluid, and ICU wards environments, among which 195 isolates were from sputum and 86 were from ICU wards environments. As for the distribution of wards, 295 isolates were mainly from ICU (277), including ICU environments. Other wards, such as NW and HCW, had a few isolates. These results demonstrated that ICU was always the ward with the most serious nosocomial infection of *A. baumannii* (10–12), indicating that regular disinfection of the ICU and air environment is necessary.

The emergence of MDR *A. baumannii* has brought great challenges to clinical treatment. Fifteen antibiotics were used for susceptibility testing in this study, and the result showed that all *A. baumannii* isolates exhibit MDR phenotypes. In terms of time axis, there was no significant change in the resistance of MDR *A. baumannii* in the hospital over the 12 years from 2008 to 2019. Generally, except for tigecycline and polymyxin E, these isolates were almost resistant to all available antimicrobial agents, including imipenem and meropenem. The main mechanism of carbapenem resistance in MDR *A. baumannii* is the acquisition of carbapenem-hydrolyzing oxacillinase-encoding genes. Of these, *bla*_*OXA*−23_ carbapenemase-resistant gene was by far the most widespread in most countries (13), and it was found that *bla*_*OXA*−23_-producing *A. baumannii* isolates disseminated widely in China or Asian (14–16). In our study, the detection of resistant genes showed that the most common carbapenemase-resistant gene was *bla*_*OXA*−23_ (288 isolates) in all *A. baumannii* isolates, followed by *bla*_*OXA*−66_ (212), which nearly could be detected in all periods except 2015–2016, indicating that these two drug-resistant genes might be the main reason for bacterial carbapenem resistance. Additionally, *bla*_*TEM*−1_ is the most largely known and classic β-lactamase. It was reported that the expression of *bla*_*TEM*−1_ β-lactamase positively correlated with the minimum inhibitory concentration of sulbactam, and transfer of the blaTEM-1 gene into a susceptible *A. baumannii* strain resulted in resistance (17). Recently, Yang et al. collected 2,197 *A. baumannii* isolates from 27 provinces in China, found that the resistance rate for cefoperazone–sulbactam was 39.7%, and demonstrated that *bla*_*TEM*−1_ with four tandem copies structure played a key role in this resistance phenomenon (18). Meanwhile, Han et al. proved that *bla*_*OXA*−23_and *bla*_*TEM*−1_genes were more conducive to resistance to carbapenems in *A. baumannii* (19). In the current study, *bla*_*TEM*−1_ gene had been detected in all isolates since 2012, and the overall positive rate of this resistance gene was 75.3% (222/295), meanwhile, the cefoperazone–sulbactam resistance rate of the 295 MDR *A. baumannii* isolates was up to 94.5%, and both had a certain correlation relationship. Further genome sequence analysis of ST451 in 2019, there was only one copy of TEM-1 in the MDR *A. baumannii* isolates in the study, indicating the specific molecular mechanism of the resistance of *A. baumannii* to cefoperazone–sulbactam might be diversity. As an important resistance gene of *A. baumannii*, only one isolate carried *bla*_*NDM*−1_ in 2010–2011, however, given its distribution worldwide, enhanced monitoring is needed (20, 21). In addition, *A. baumannii* isolates carried several aminoglycoside-resistant genes and gene for 16s rRNA methylation enzymes, of which *ant(3")-I* and *armA* could be detected every year. Overall, these drug-resistant genes should be further monitored to understand their structure and analyze the characteristics of drug resistance.

It has been reported that ST208 and ST195 were the predominant epidemic types of MDR *A. baumannii* in China (22, 23). In this study, the STs of *A. baumannii* isolates have undergone a significant change over the past 12 years, the epidemic STs changed from ST208 and ST369 at the early stage (2008–2016) to the predominant ST451 in 2019 after the transition of ST195 (2017–2018). Lee et al. reported that the outbreak of XDR *A. baumannii* ST451 carrying MDR genes occurred in South Korea (24). Moreover, there have been outbreaks of *A. baumannii* ST451 in countries around China in recent years, such as Thailand and India (25–27), and ST451 was also isolated from patients with bloodstream infection, causing a certain mortality (28), so the prevalence of *A. baumannii* ST451 requires more attention. In order to understand the molecular characteristic and the transmission route of ST451 outbreak, one ST451 (Ab1) isolated from the environment and another ST451 (Ab2) from a patient in 2019 were selected for the genome sequence (Supplementary Figure 2). The results showed that the average nucleotide identity of both isolates was >99.9%, indicating that the ST451 MDR *A. baumannii* that caused an outbreak in 2019 were the same clone. Therefore, although there were 59 ST451 *A. baumannii* isolates, all isolates may be the same clone. Moreover, the STs of MDR *A. baumannii* isolates changed, but the antibiotic susceptibility of ST451 isolates did not change significantly, indicating that there was no significant relationship between the STs of the isolates and drug resistance. We thought that the drug-resistant phenotypes of bacteria were mainly related to the drug-resistant genes. According to eBURST analysis, ST451 and ST208 belonged to Group 1, and they had a close genetic relationship. The detection also showed that the drug-resistant genes carried by ST451 were similar to those carried by ST195 and ST208 earlier. The prevention and control strategy adopted by the hospital was to remove all patients from the wards for disinfection, and to disinfect or replace the medical supplies used by the patients. Moreover, we also found that MDR *A. baumannii*–infected patients with respiratory tract infection were easy to pollute the surrounding environments. When the bed sheets and pillowcases of patients were seriously polluted, MDR *A. baumannii* could be detected within 1.5 m when patients turned over. Therefore, the safe distance between patients should be increased to 3 m as far as possible to prevent cross-infection.

On the whole, the resistance and resistant genes of the prevalent MDR *A. baumannii* isolates in the hospital have not changed significantly over the past 12 years. However, almost all MDR *A. baumannii* isolates carried multiple antibiotic-resistant genes, and the predominant MDR *A. baumannii* isolates have evolved from ST208 and ST369 to ST451. Therefore, it is of great significance to further strengthen the epidemiological surveillance of *A. baumannii*, analyze its genetic evolution, and provide new strategies for the prevention and control of nosocomial infections caused by MDR *A. baumannii*.

## Data Availability Statement

The original contributions presented in the study are included in the article/Supplementary Material, further inquiries can be directed to the corresponding authors.

## Ethics Statement

The studies involving human participants were reviewed and approved by The Ethics Committee of Taian City Central Hospital. The patients/participants provided their written informed consent to participate in this study. All the subjects gave a written informed consent in accordance with the Declaration of Helsinki.

## Author Contributions

MJ and XC performed the main experiments, analyzed data, and wrote the manuscript. SL, ZZ, NL, CD, and LZ performed the experiment and analyzed data. HW and SZ reviewed the manuscript and approved it. All authors contributed to the article and approved the submitted version.

## Conflict of Interest

The authors declare that the research was conducted in the absence of any commercial or financial relationships that could be construed as a potential conflict of interest.

## Publisher's Note

All claims expressed in this article are solely those of the authors and do not necessarily represent those of their affiliated organizations, or those of the publisher, the editors and the reviewers. Any product that may be evaluated in this article, or claim that may be made by its manufacturer, is not guaranteed or endorsed by the publisher.
