# Supplementary material for: Epidemiological Analysis of Multidrug-Resistant Acinetobacter baumannii Isolates in a Tertiary Hospital Over a 12-Year Period in China
Source: Front Public Health. 2021 Aug 12;9:707435. doi: 10.3389/fpubh.2021.707435 (PMC8388840; doi:10.3389/fpubh.2021.707435)
Supplement: Supplementary file 1 [file Data_Sheet_1.docx]

**Supplementary materials**

Table S1. The detail information of 86 MDR A. baumannii isolates from patients and wards environments in 2018-2019

| **Period** | **Ward distribution^a^** | **Samples origin** | **ST** |
| --- | --- | --- | --- |
| 2018 | GW | Cerebrospinal fluid of patient 1 | 451 |
|  | GW | Sputum of patient 1 | 451 |
|  | NSW | Sputum of patient 2 | 369 |
|  | NSW | Cerebrospinal fluid of patient 2 | 369 |
|  | ICU1 | Cerebrospinal fluid of patient 3 | 451 |
|  | ICU1-E | On-line hemofiltration used by patient 3 | 451 |
|  | ICU1-E | Injection pump used by patient 3 | 451 |
|  | ICU1-E | Quilt surface used by patient 3 | 451 |
|  | ICU1-E | Desk used by patient 3 | 451 |
|  | ICU1-E | Pillow slips used by patient 3 | 451 |
|  | ICU1-E | Monitor used by patient 3 | 451 |
|  | ICU1 | Sputum of patient 4 | 195 |
|  | ICU1-E | The right bed handrail used by patient 4 | 1176 |
|  | ICU1-E | Desk used by patient 3 | 195 |
|  | ICU3 | Cerebrospinal fluid of patient 5 | 195 |
|  | ICU3 | Sputum of patient 5 | 195 |
|  | ICU3-E | Desk used by patient 5 | 195 |
|  | ICU3-E | Pillow slips used by patient 5 | 195 |
|  | ICU3-E | Quilt surface used by patient 5 | 195 |
|  | ICU3-E | The button of injection pump used by patient 5 | 195 |
|  | ICU3-E | Tube of ventilator used by patient 5 | 195 |
|  | ICU3-E | The lower bed handrail used by patient 5 | 195 |
|  | ICU3-E | Injection pump used by patient 5 | 195 |
|  | ICU3-E | The right bed handrail used by patient 5 | 195 |
|  | ICU3-E | Button of bed used by patient 5 | 195 |
|  | ICU3-E | The surface of respiratory monitor used by patient 5 | 195 |
|  | ICU3-E | Monitor used by patient 5 | 195 |
| 2019 | ICU3 | Sputum of patient 6 | 451 |
|  | ICU3-E | The surface of respiratory monitor used by patient 6 | 451 |
|  | ICU3-E | Bed sheet used by patient 6 | 451 |
|  | ICU3-E | Quilt cover used by patient 6 | 451 |
|  | ICU3-E | Bedside rail restraint used by patient 6 | 451 |
|  | ICU3-E | The surface of injection pump used by patient 6 | 451 |
|  | ICU3-E | Hand disinfector used by patient 7 | 451 |
|  | ICU3-E | Distillation bottle used by patient 7 | 451 |
|  | ICU3-E | Quilt cover used by patient 7 | 451 |
|  | ICU3-E | Clothes used by patient 7 | 451 |
|  | ICU3 | Sputum of patient 7 | 451 |
|  | ICU3-E | Bed sheet used by patient 7 | 451 |
|  | ICU3 | Sputum of patient 8 | 451 |
|  | ICU3-E | Clothes used by patient 8 | 451 |
|  | ICU3-E | Quilt cover used by patient 8 | 451 |
|  | ICU3-E | Armbands for sphygmomanometer used by patient 8 | 451 |
|  | ICU3-E | Stethoscope used by patient 8 | 451 |
|  | ICU3-E | The surface of nightstand used by patient 8 | 451 |
|  | ICU3-E | The bed handrail used by patient 8 | 451 |
|  | ICU3-E | The tube of ventilator used by patient 8 | 451 |
|  | ICU3 | Sputum of patient 9 | 451 |
|  | ICU3-E | Distillation bottle used by patient 9 | 451 |
|  | ICU3 | Sputum of patient 10 | 451 |
|  | ICU3-E | The nightstand used by patient 10 | 451 |
|  | ICU3-E | Armbands for sphygmomanometer used by patient 10 | 451 |
|  | ICU3-E | Nurse’s cuff of nursing patient 10 | 451 |
|  | ICU3-E | Bedside rail restraint used by patient 10 | 451 |
|  | ICU3-E | The button of ventilator used by patient 10 | 451 |
|  | ICU3-E | The tube of ventilator used by patient 10 | 451 |
|  | ICU3-E | Ventilator used by patient 10 | 451 |
|  | ICU3-E | Pillow slips used by patient 10 | 451 |
|  | ICU3 | Sputum of patient 11 | 451 |
|  | ICU3-E | The button of left bed handrail used by patient 11 | 451 |
|  | ICU3-E | The button of right bed handrail used by patient 11 | 451 |
|  | ICU3-E | The left bed handrail used by patient 11 | 451 |
|  | ICU3-E | The right bed handrail used by patient 11 | 451 |
|  | ICU3-E | Distillation bottle used by patient 11 | 451 |
|  | ICU3-E | Doctor cuffs of treating patient 11 | 451 |
|  | ICU3-E | Nurse cuffs of nursing patient 11 | 451 |
|  | ICU3-E | The left wristband used by patient 11 | 451 |
|  | ICU3-E | The right wristband used by patient 11 | 451 |
|  | ICU3-E | Sphygmomanometer used by patient 11 | 451 |
|  | ICU3-E | The head of bed used by patient 11 | 451 |
|  | ICU3-E | The foot of bed used by patient 11 | 451 |
|  | ICU3-E | Quilt used by patient 11 | 451 |
|  | ICU3-E | Pillow used by patient 11 | 451 |
|  | ICU3-E | Desk used by patient 11 | 451 |
|  | ICU3-E | Ventilator used by patient 11 | 451 |
|  | ICU3-E | Infusion support used by patient 11 | 451 |
|  | ICU3 | Sputum of patient 12 | 451 |
|  | ICU3 | Sputum of patient 13 | 451 |
|  | ICU3 | Sputum of patient 14 | 451 |
|  | ICU3-E | Air around patient 13 | 451 |
|  | ICU3-E | Air around patient 5 | 191 |
|  | ICU3-E | Air around patient 14 | 451 |
|  | ICU3-E | Desk in the nurse station | 451 |
|  | ICU3-E | Printer in the nurse station | 451 |
|  | ICU3-E | Hook of the small car | 451 |
|  | ICU3-E | The mouse in nurse station | 451 |

^a^ GW, Gastroenterology ward; NSW, Neurosurgery ward; ICU, Intensive care unit; ICU1-E, ICU1 environment; ICU3-E, ICU3 environment.


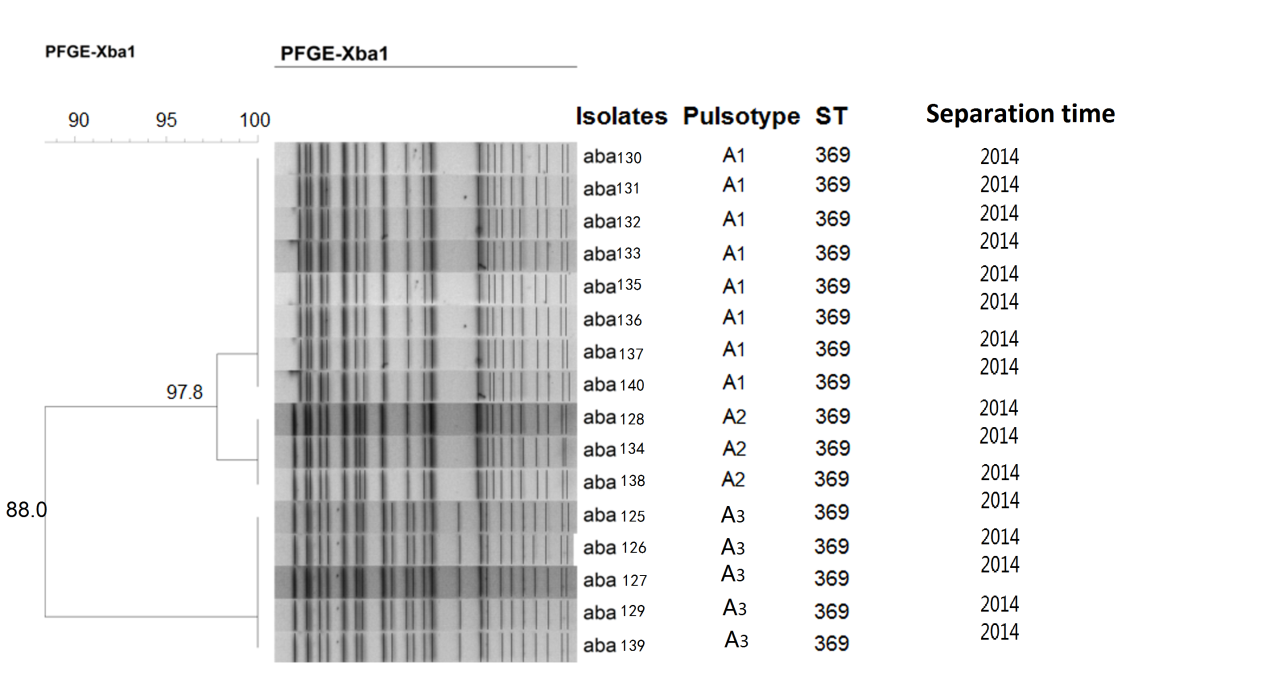


**A**


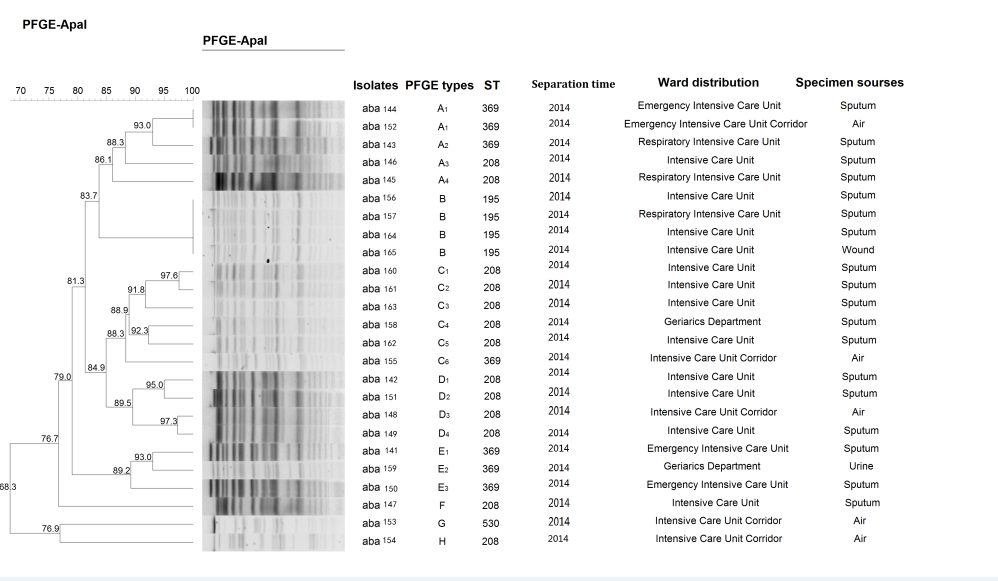


**B**


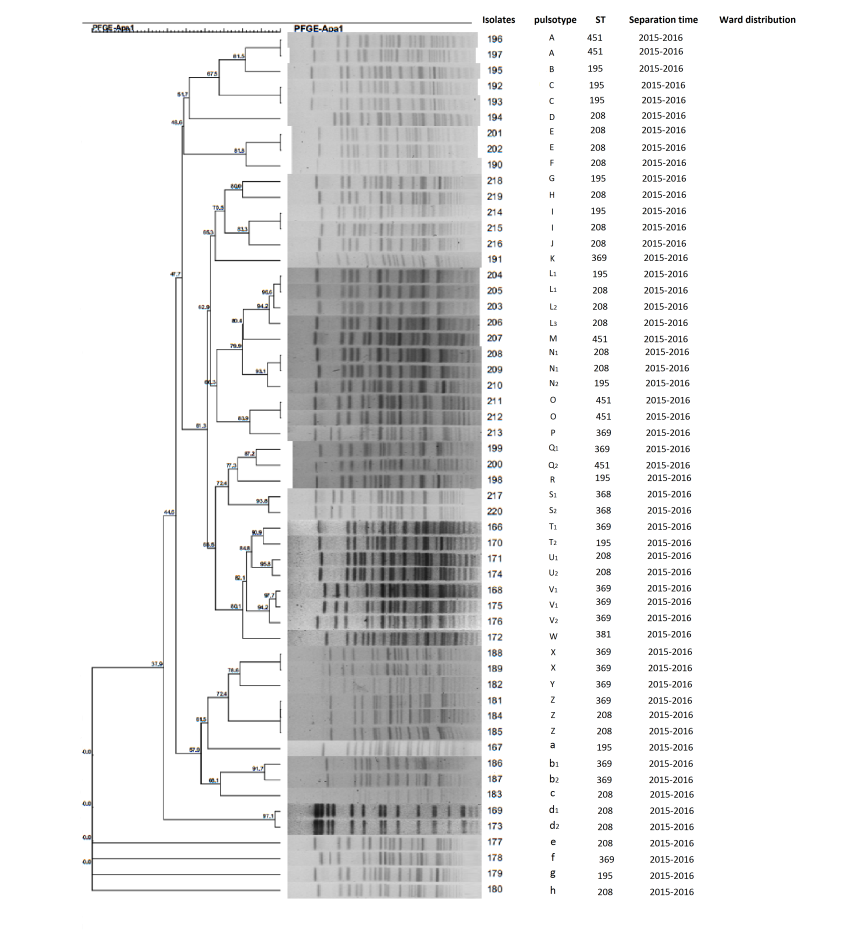


**C**

Figure S1. The PFGE results of MDR *A. baumannii* isolates obtained from 2014 to 2016. (A) Sixteen MDR *A. baumannii* isolates from January 31, 2014 to February 1, had only one PFGE type (type A). (B) Twenty-five MDR *A. baumannii* isolates from May 2014 to November 2014, had 8 types (A-H), including 6 type C, 5 type A, 4 type B and D, respectively. (C) Fifty-five MDR *A. baumannii* isolates obtained in 2015-2016 constituted 34 PFGE types, among them, type L had 4 isolates, type N, V, and Z individually had 3 isolates.


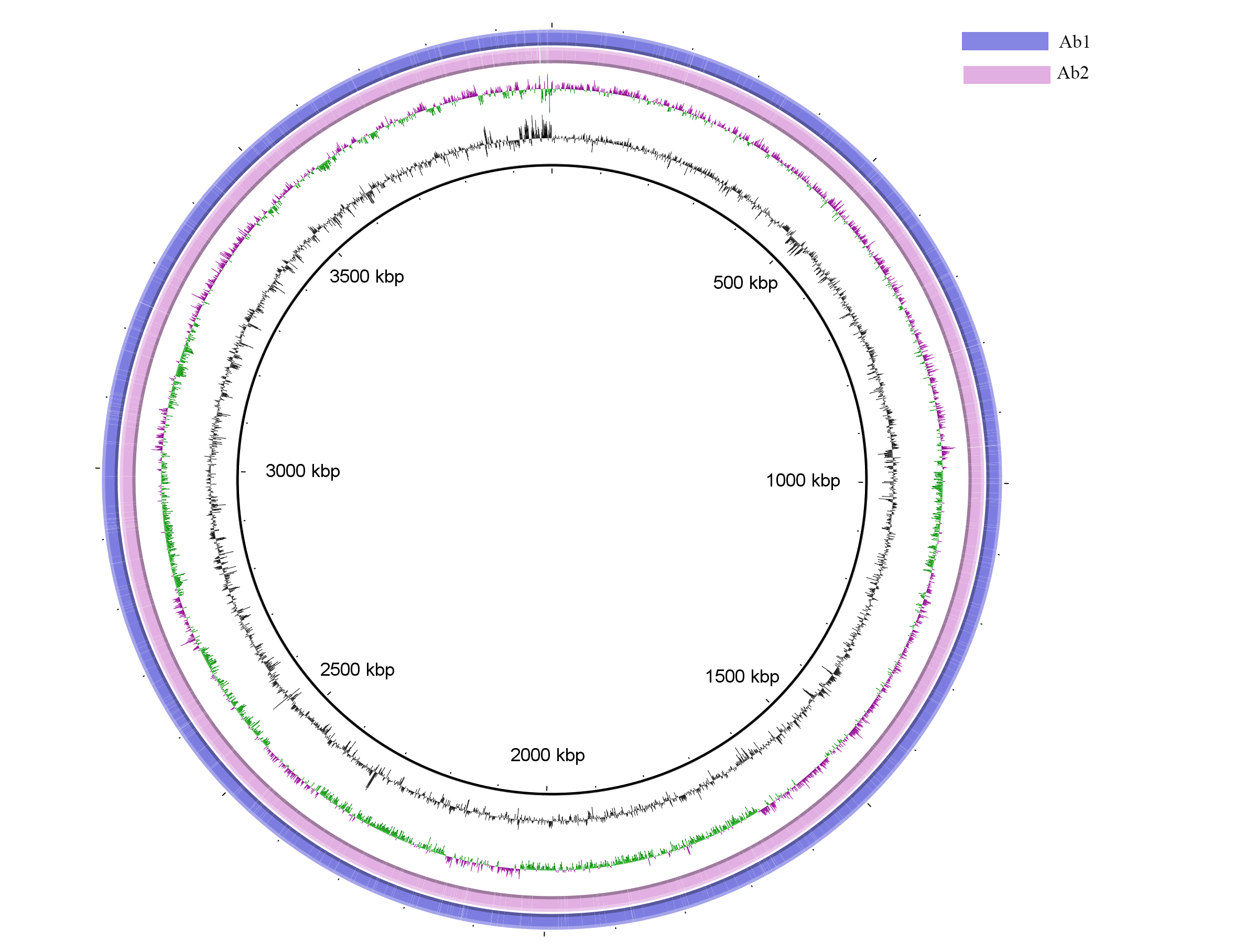


Figure S2. The genome sequence result of the representative ST451 isolates in 2019. Ab1 was isolated from the environment and Ab2 from a patient.
